# A Japanese LDA model for automatic clustering analysis of semantic verbal fluency tests

**DOI:** 10.3758/s13428-025-02696-1

**Published:** 2025-06-30

**Authors:** Masahiro Yoshihara, Yoshihiro Itaguchi

**Affiliations:** 1 Graduate School of International Cultural Studies, Tohoku University, Sendai, Japan; 2 Department of Psychology, Keio University, 2-15-45, Mita, Minato-ku, Tokyo, 108-8345 Japan

**Keywords:** Verbal fluency test, Semantic clusters, Latent Dirichlet allocation, Japanese

## Abstract

In the semantic variant of verbal fluency tests (VFTs), clustering analysis has become popular for examining the semantic structure. While the computational psycholinguistics approach has recently drawn attention to increasing the reproducibility of clustering analysis, such an approach is not available in all languages. To make the computational approach available in the Japanese language, we constructed a Japanese latent Dirichlet allocation (LDA) model. Our LDA model enables researchers and clinicians to objectively quantify the associative relationships of words, thereby making it possible to automatically detect semantic clusters. We conducted the semantic VFT with healthy young Japanese adults to examine the validity of our LDA model. We performed clustering analyses using the computational approach with our LDA model and the conventional manual approach with human coders. The results showed that the LDA model identified semantic clusters, as did the human coders. In addition, we demonstrated for the first time that response intervals within a cluster were significantly shorter than those outside of clusters, regardless of the clustering approaches. This indicates that both approaches reflect a broadly accepted assumption that closer semantic relations require less processing time. However, LDA-based clustering produced, on average, larger clusters than human-based clustering did, indicating that the LDA model captured semantic relationships between words that human coders would not recognize. Taken together, the present results demonstrated the validity of our LDA model. We hope that our LDA model fosters the use of the computational linguistic approach in semantic VFTs with Japanese participants.

**Supplementary Information:**

The online version contains supplementary material available at 10.3758/s13428-025-02696-1.

## Introduction

In cognitive sciences, semantics is one of the most important (and difficult) research topics. To unveil the nature of semantic systems, researchers have used various tasks. One such task is a semantic verbal fluency test (a semantic VFT), in which participants are asked to produce as many words as possible that belong to a given semantic category (e.g., the category “animal”) within a time limit (e.g., 1 min). Semantic VFTs have been widely used in academic and clinical settings to assess the cognitive ability to retrieve semantic information without errors (e.g., Ardila, 2020; Gomez & White, 2006; Henry et al., 2004; Itaguchi et al., 2022; Loewenstein et al., 2012; Villalobos et al., 2022). Specifically, data from semantic VFTs are valuable because they can be used to diagnose cognitive impairments (Lezak, 2005). For instance, the total number of correct responses was significantly lower in patients with Alzheimer’s disease (AD) than in healthy controls (Gomez & White, 2006). Similarly, scores on the semantic VFT may be a predictor of mild cognitive impairment (MCI; Loewenstein et al., 2012). Previous literature has also indicated the relationships between the performance in the semantic VFT and various clinical populations, including individuals with aphasia (Bose et al., 2017), frontal-lobe lesions (Troyer et al. 1998a, b), Parkinson’s disease (Tröster et al., 1998; Troyer et al. 1998a, b), Huntington’s disease (Rich et al., 1999), schizophrenia (Robert et al., 1998), and brain injury (Engstad et al., 2003; Henry & Crawford, 2004; Schmidt et al., 2019; Woods et al., 2016).

Although semantic VFTs are useful, there is an important question in the literature: What measure should be analyzed? The most common and conventional measure is the total number of correct responses (Acevedo et al., 2000; Kozora & Cullum, 1995; Tröster et al., 1989, 1998). However, this quantitative measure has been criticized because it provides little insight into the underlying mechanisms of semantic VFTs. Analysis of the total correct responses does not reveal either how healthy participants fluently produce semantically related words or why some patients show impairments in this task (e.g., Itaguchi et al., 2022; Troyer, 2000; Troyer et al., 1997). Therefore, qualitative measures have also been used in the literature to further investigate the processes underlying semantic VFTs. For instance, several researchers pointed out the importance of analyzing error responses (e.g., Auriacombe et al., 2006; Itaguchi et al., 2022; Troyer, 2000), the exact timing of responses (Holmlund et al., 2019) and word frequency counts of response words (Adams et al., 1989; Crowe, 1998; Daimon et al., 2021). Furthermore, and more relevant to the present study, Troyer and colleagues (e.g., 1997, 2000) suggested that the analysis of semantic clusters and switches is highly informative for understanding the mechanisms of semantic VFTs.

Semantic clusters are consecutive responses that are related to the same subcategories (e.g., “pets”, “farm animals”) and switches are shifts between clusters (Rofes et al., 2020; Troyer et al., 1997; Troyer et al. 1998a, b). For example, when a participant produces a sequence of “dog, cat, parakeet, cow, sheep,” the first three responses constitute one cluster (e.g., “pets”), and the last two responses constitute another cluster (e.g., “farm animals”). When response words in the first subcategory are exhausted, the participant switches to the second subcategory. In the literature, clustering is related to temporal-lobe functions involving the identification of semantic subcategories (e.g., Troyer et al. 1998b) and access to lexical/semantic representations in the subcategories (e.g., Bose et al., 2017). On the other hand, switching is related to frontal-lobe functions such as cognitive flexibility (e.g., manipulations of attention: Troyer et al. 1998b; initiating a new lexical search process when a cluster is exhausted: Troyer, Moscovitch, Winocur, Alexander et al. 1998a). In addition, researchers have examined the relationships between the two measures (i.e., clusters and switches) and working memory: switching is related to processing speed, whereas clustering is related to vocabulary (e.g., Bushnell et al., 2022; Unsworth et al., 2011). As such, analysis of clusters and switches allows us to tap into the semantic processing underlying the VFT.

Specifically, clusters are assumed to reflect the semantic structure of a certain category represented in a participant (e.g., Rosenstein et al., 2015). For instance, constituents of a semantic cluster are quite different across participants, potentially reflecting that people show divergence in the structure of their semantic network (e.g., Mollo et al., 2016; Rosenstein et al., 2015). Furthermore, the analysis of clusters is informative in terms of clinical diagnosis. Previous literature revealed that the number of clusters and the mean cluster size (MCS; i.e., the average number of responses within a cluster) were smaller in patients with (established) AD than in healthy controls (e.g., Fagundo et al., 2008; Rosen, 1980; Tröster et al., 1989; Troyer et al. 1998a, b; but see Raoux et al., 2008). Likewise, previous literature has reported smaller MCS, compared to healthy controls, in patients with (demented) PD or Huntington’s disease (e.g., Tröster et al., 1998; cf. Troyer et al. 1998b) or aphasia (e.g., Bose et al., 2017). These results suggest functional disturbances in semantic structure in these clinical populations. As such, clustering analysis provides detailed insights into the semantic processes underlying VFTs.

While previous literature demonstrated that semantic cluster analysis is useful to tap into the semantic structure, there are methodological concerns. For instance, as several researchers have noted (Itaguchi et al., 2022; Pakhomov et al., 2015; Pakhomov & Hemmy, 2014; Zemla et al., 2020), semantic clusters are often manually determined based on subjective assessments of a researcher or clinician. Such subjectivity is problematic because the decision criteria would be inconsistent across studies. To address the subjectivity of semantic clustering, some researchers compare clustering results between multiple coders, and calculate reproducibility indices (e.g., kappa coefficients or intraclass correlation coefficients). However, clustering results are not perfectly reproducible because the congruency between coders is generally not 100%. It is thus difficult, if not impossible, to directly compare the results across previous studies of semantic VFTs. Another concern is that the manual assessment of clusters is often time-consuming and labor-intensive (Gomez & White, 2006; König et al., 2018; Zemla et al., 2020). This may hinder the application of semantic cluster analysis, especially in clinical settings, where therapists typically do not have the time to perform manual clustering analysis.

As a solution to these methodological concerns, several researchers have suggested that a computational linguistic approach would be effective for detecting semantic clusters (Holmlund et al., 2019; Itaguchi et al., 2022; Pakhomov et al., 2015; Pakhomov & Hemmy, 2014; Rosenstein et al., 2015). Computational models automatically produce objective results of semantic clustering. Pakhomov and Hemmy (2014), for instance, proposed a novel approach using latent semantic analysis (LSA; e.g., Deerwester et al., 1990; Landauer & Dumais, 1997) to identify semantic clusters. LSA is a technique for quantifying the semantic content of words based on the co-occurrence of words in a large corpus of documents. This technique helps us to assess the semantic relatedness between two words, which makes it possible to automatically detect semantic clusters. Pakhomov and Hemmy, for the first time, used LSA in semantic cluster analysis. In evaluating their approach, they investigated whether LSA-based analysis could predict the risk of dementia. Using a dataset from a large-scale longitudinal aging study (i.e., the Nun study; e.g., Snowdon, 1997), they demonstrated that clustering measures (e.g., MCS) computed using LSA indeed worked as significant predictors of dementia risk. Their findings thus suggest that the computational linguistic approach is useful.

Furthermore, Itaguchi et al. (2022) proposed latent Dirichlet allocation (LDA; e.g., Blei et al., 2003) as an alternative approach for objective and automatic clustering analysis. LDA is one of the most popular natural language processing methods (Jelodar et al., 2019), which assumes that multiple latent topics coexist in a document. LDA extracts them by calculating the probability of co-occurrence of words. The generalizability of the latent topics obtained by LDA is usually greater than similar probabilistic approaches, such as latent semantic analysis (LSA) and explicit semantic analysis (ESA). LDA also has an advantage over modern neural network techniques such as Word2 Vec (Mikolov et al., 2013a, b), GloVe (Pennington et al., 2014), and BERT (Devlin et al., 2019) in the interpretability of the results, because the extracted topics in LDA correspond to the concept of subcategory in semantic VFTs. Therefore, LDA is suitable as a computational linguistic approach for semantic cluster analysis.

In a VFT using the category “animal” with Norwegian, Itaguchi et al. (2022) showed that the LDA-based approach can be particularly useful for identifying semantic clusters in errors that have been neglected in human coding. Specifically, they conducted semantic cluster analysis using topic probabilities in their LDA model. Topic probability quantifies how likely a latent topic exists when a specific word is used in a document. Assuming that topics in LDA correspond to subcategories (e.g., “pets”) of a given category (e.g., “animal”), they automatically detected a cluster if two consecutive responses in the VFT had a topic probability above a threshold for the same topic. Furthermore, they showed that the LDA-based cluster analysis revealed latent semantic structures. That is, although patients with mild AD produced more intrusion errors (i.e., words that do not belong to a given category) than healthy controls, these intrusion errors were potentially produced by association with a previous response; for example, an intrusion error, “veterinarian” was produced after the responses “deer, fallow deer”, which constituted a cluster. Itaguchi et al. found that approximately 60% of the intrusion errors occurred within semantic clusters. These results suggest that while the organization of the semantic network deteriorates in individuals with mild AD, semantic associations are still preserved to some extent. The authors argued that this finding would not be found if a human coder manually analyzed the semantic clusters: Human coders would *not* consider intrusion errors to be related to the “animal” category because the error responses were literally defined as “non-animal” words. Thus, the LDA-based approach is useful in semantic cluster analysis.

Unfortunately, although previous literature has shown the fruitfulness of computational linguistic approaches (e.g., Holmlund et al., 2019; Itaguchi et al., 2022; Pakhomov & Hemmy, 2014; Pakhomov et al., 2015; Rosenstein et al., 2015), this approach is not available for all languages. To the best of our knowledge, computational linguistic models have been implemented for the semantic VFTs with Roman alphabetic languages only (German: Hähnel et al., 2023; Norwegian: Itaguchi et al., 2022; English: Pakhomov & Hemmy, 2014). However, it is inappropriate to apply these models to a semantic VFT performed in other languages because the performance of computational linguistic approaches (e.g., LSA, LDA) highly depends on the language of a corpus that is used to construct the computational models. Furthermore, participants’ languages and cultures may have different effects on response strategies in VFTs (e.g., Ardila, 2020; Kempler et al., 1998; Mitsuto et al., 2019). For instance, linguistic factors such as word length (Kempler et al., 1998) and bilingualism (Ardila, 2020) may differentially affect the results of VFT across languages. In addition, participants may produce animal names that are specific to the culture in which they are born. For example, native Japanese participants may tend to produce the names of animals that reside in the Japanese “satoyama” landscape (i.e., undeveloped woodland near a village that is specific to Japan; e.g., Mitsuto et al., 2019). Thus, it is important to implement a computational linguistic model that is tuned to non-alphabetic languages such as Japanese.

In the present study, we provide a Japanese LDA model to promote the application of the computational linguistic approach to semantic VFTs with Japanese participants or patients. In addition, we conducted a semantic VFT with healthy Japanese young adults and analyzed the data using both the computational linguistic approach with our Japanese LDA model and the conventional manual approach. By comparing the cluster-related measures (e.g., MCS) between these two approaches, we evaluate the validity of our LDA-based approach. Specifically, we investigate whether our Japanese LDA model would identify semantic clusters as the manual approach does.

To further evaluate the validity of our LDA approach from a different perspective, we also examined the relationships between time intervals of verbal responses and semantic clusters (e.g., Bose et al., 2017; cf. Gruenewald & Lockhead, 1980). Specifically, we tested whether time intervals of consecutive responses within a cluster (within-cluster interval; WCI) are shorter than those outside clusters (out-of-cluster interval; OCI). This prediction was derived from a widely accepted assumption that closer semantic relations require less processing time (Collins & Loftus, 1975; Holmlund et al., 2019; Neely, 1991). For instance, it is well known that word-nonword discrimination of a target word (e.g., “doctor”) is easier when it is preceded by a semantically related word (e.g., “nurse”) than when it is preceded by an unrelated word (e.g., “bread”; i.e., the semantic priming effect; see Neely, 1991, for a review). Analogously, when an animal word is produced in the semantic VFT, another word that is semantically related to that word would not take long to be produced successively (and they would constitute a semantic cluster; i.e., WCI). On the other hand, it would take some time to produce semantically unrelated words (and they would not constitute a cluster; i.e., OCI). If our LDA model captures these relationships, we would observe a shorter WCI compared to the OCI.

## Methods

### LDA model

In the current study, we chose LDA, rather than LSA, as a suitable approach for automated clustering in VFTs because LDA is known to outperform LSA (e.g., Griffiths & Steyvers, 2004). In addition, LDA is more flexible than LSA. As LDA is a Bayesian inference model, it can be generalized to new data relatively easily (e.g., Blei et al., 2003). Thus, LDA is thought to have advantages over LSA, and hence LDA has been used in psycholinguistic research (e.g., Hofmann et al., 2022; Pereira et al., 2013). Therefore, we decided to use LDA in the present study.

### Stimuli

We used the Japanese version of the Wikipedia database (jawiki-20181020-pages-articles.xml.bz2, 1,125,721 articles) as a dataset to construct an LDA model. However, instead of using all articles, we focused on articles related to the category “animal” because this is the most frequently used category in the literature on semantic VFTs (Acevedo et al., 2000; Itaguchi et al., 2022; Villalobos et al., 2022). We targeted 661 Japanese animal names collected from the various websites of zoos and aquariums in Japan. These animal names appeared in more than ten Wikipedia articles. We then extracted articles that referred to at least one of these animal names. As a result, the dataset was reduced to 24,737 articles containing animal names.

### The number of topics and topic probabilities

We performed LDA analysis on the dataset using the Rstan package (version 2.18.2, Stan Development Team, 2018) available in R (version 3.5.2). The LDA model was estimated with parameters of the number of topics = 10 and alpha = 0.4. We determined the number of topics based on the interpretability of the resulting topics and the generalization performance to a novel task (i.e., the semantic VFT in the current study; e.g., Steyvers et al., 2006). After trying a range of values (i.e., 5, 10, 15, 20) in the estimation of the LDA models, we found that the model with ten topics was the most interpretable. In other words, topics in models with fewer or more topics were too broad or idiosyncratic. Given that the number of topics identified by human coders was also around ten in previous literature (e.g., Fagundo et al., 2008; Itaguchi et al., 2022; Mitsuto et al., 2019; Troyer et al., 1997), ten topics would be preferable when investigating the semantic structure of “animal” category. In addition, as discussed later, our LDA model successfully identified semantic clusters in the semantic VFT of healthy Japanese young adults. Note that we also determined the value of another parameter, alpha (i.e., 0.4), which is assumed to reflect the number of topics per document (e.g., Panichella, 2021), considering the interpretability of the results.

Using the LDA model estimated with these parameters, we then computed topic probability. As noted in the Introduction, this is the probability that a latent topic exists when a particular word (e.g., an animal name, “dog”) is used in a document. In the LDA analysis, each word is assumed to have multiple topic probabilities (e.g., the word “dog” can be used when the topic is “pets” or “mammals”). Since the present model had ten topics, each word had ten topic probabilities. We assumed that these topics correspond to subcategories defined in conventional (i.e., manual) semantic cluster analysis (Fagundo et al. 2008; Itaguchi et al., 2022). Thus, a topic probability represents the probability that an animal name is related to a subcategory. The sum of the ten topic probabilities for a word is 1 (i.e., 100%). Examples of animal names in each topic are shown in Table 1. The full list of animal names and their topic probabilities is uploaded to OSF (see Supplementary Data File 1).

**Table 1:** Examples of animal names in each of the ten topics

Topic ID	Examples of animal names
1	Poodle, Oriental stork, Baby Bat, Mongoose, Japanese white-eye, Prairie Dog, Japanese bush warbler, Japanese Serow, Marmot, Sika deer
2	Carp, Sardine, Horse mackerel, Crucian carp, Sweetfish, Japanese sea bass, Largemouth Bass, Loach, Mackerel, Swordfish
3	Toothed Whale, Cobra, Yezo sika deer, Sea snake, Bottlenose Dolphin, Northern goshawk, Roof Rat, Dolphin, Ezo red fox, Cookiecutter shark
4	Lizard, Iguana, Chameleon, Crocodile, Snake, Cat, Frog, Rockhopper penguin, Mouse, Camel
5	Pig, Cow, Water buffalo, Horse, Chicken, Gazelle, Sheep, Eastern quoll, Bear, Goat
6	Zebra, Giraffe, Rail, Elephant, Hyena, Ostrich, Rhinoceros, Brown bear, Bison, Hippopotamus
7	Fin whale, Humpback whale, Minke whale, Right whale, Elephant Seal, Sperm Whale, Blue whale, Harbor seal, Steller sea lion, Gorilla
8	Rat, Parakeet, Parrot, Human, Degu, African Grey Parrot, Goat, Monkey, Rabbit, European rabbit
9	Wild boar, Swan, Rabbit, Eagle, Sheep, Deer, Cow, Dog, Quail, Horse
10	Turkey, African elephant, Quail, Shepherd, Duck, Chihuahua, Labrador Retriever, Asian elephant, Topi, Sea lion

The examples are animal names that had the top ten highest topic probabilities in each topic. Because all animal words have topic probabilities for the ten topics, it is not appropriate to assume that each belongs to only one of the topics. The Topic IDs do not correspond directly to conventional subcategories such as “pets” and “farm animals,” and the order of the IDs has no specific meaning

### Verbal fluency task

#### Participants

Forty-nine undergraduate and graduate students from Waseda University participated in this experiment (age: 20.6 years on average, *SD* = 1.3, number of women: 30). They were paid for their participation. All were native Japanese speakers with normal or corrected-to-normal vision. They reported no history of neurological disorders or traumatic brain injury that could affect cognitive function. The present study was approved by the Ethics Review Committee on Research with Human Subjects of Waseda University (Protocol # 2018-051).

#### Apparatus and procedure

The participants were tested individually in a quiet room. The experiment was programmed using MATLAB 2018a (MathWorks, Inc.). The participants were seated in front of a computer screen. The experiment began with the presentation of the category name “animal” in Japanese. The participants were then asked to verbally produce as many animal names as possible within one minute. They were instructed not to repeat the same animal names. Participants’ responses were recorded on a PC. Prior to the experimental trials, participants were given examples of words belonging to the category “home electric appliances” to familiarize them with the task.

#### Identification of clusters

We identified clusters using two different approaches: an automatic approach and a manual approach. In the automatic approach, we used the topic probabilities computed by the LDA model to identify clusters. A cluster was detected when two consecutive responses had topic probabilities that exceeded a criterion (i.e.,.13) on the same topic. This criterion was determined by considering the lowest value of the maximum topic probabilities among the words in our LDA model: The maximum topic probability of an animal name, yellow tang, was.136. This value was the lowest maximum topic probability of the animal names used in our LDA model. The criterion was set at.13 to allow any word to form a cluster. It is important to note that because a word has multiple topic probabilities (i.e., ten topic probabilities in our LDA model), more than one topic probability can exceed the criteria in a sequence of consecutive responses. Accordingly, unlike conventional manual clustering analyses (e.g., Gomez & White, 2006; Mitsuto et al., 2019), it was possible for a cluster to consist of words with multiple topics (Itaguchi et al., 2022). For example, in the sequence of responses “shark, dolphin, whale, elephant,” the first three responses (i.e., “shark, dolphin, whale”) constitute the topic “marine life,” whereas the last three responses (i.e., “dolphin, whale, elephant”) constitute another topic, “mammals.” Because the words “dolphin” and “whale” have overlapping topics (i.e., “marine life” and “large mammals”), the entire sequence forms a single cluster. Our LDA approach allows this kind of Markovian clustering (Blei et al., 2003): words can be used in different topics depending on the context. Therefore, the automatic LDA approach would quantify semantic associations between responses in the semantic VFT in a more realistic way.

In the manual approach, participants’ responses were clustered by two native Japanese coders. The first coder (coder 1) was a professional speech therapist who was naïve to the purpose of the present study. The second coder (coder 2), who was not a therapist, was the first author. The coders judged whether consecutive responses were semantically associated based on their subjective ad hoc judgment. The coders were instructed *not* to consider their own (idiosyncratic) subcategories to which the consecutive responses might be related. Instead, they were asked to focus on associative relatedness between consecutive responses. Semantic clustering was thus based on a similar procedure (i.e., focusing on associations of consecutive responses, rather than subcategories) between the automatic and manual approaches. This setup allowed us to make convincing comparisons between the two approaches (i.e., manual vs. LDA). Note that all responses (including errors) were analyzed by both LDA and manual clustering to elucidate semantic relationships over time.

#### VFT measures

To evaluate the two approaches to semantic cluster analysis, we calculated the following measures: mean cluster size (MCS; Troyer et al., 1997), within-cluster interval (WCI), and out-of-cluster interval (OCI). WCI is the average time interval between consecutive word responses within a cluster, whereas OCI is the average time interval between consecutive word responses that do not compose the same cluster. We computed WCI and OCI as follows. First, the first author manually determined the onset of each response using the open-source software Audacity (Audacity Team, 2021). Next, time intervals were computed for all two consecutive responses, by calculating the differences between each response onset in each participant. Finally, WCI and OCI were computed by averaging the time intervals within or outside a cluster, based on the results of each semantic clustering approach (i.e., LDA, human coder 1, and human coder 2).

In addition to the cluster-related indices, we reported conventional measures: the total number of correct responses, the number of errors (i.e., repetitions and intrusions). We also estimated the frequency effect, which was calculated by computing a regression coefficient of the logarithmic word frequency of the responses against the time (i.e., onset) at which the responses were produced during the VFT (Daimon et al. 2021). A negative coefficient of the frequency effect indicates that the participant tends to produce high-frequency words first, followed by lower-frequency words. Although these conventional measures, including the frequency effect, are not directly related to semantic clusters, we reported them given their importance in the literature on VFTs.

## Results and discussion

### Japanese LDA model of the animal category

**Fig. 1:**
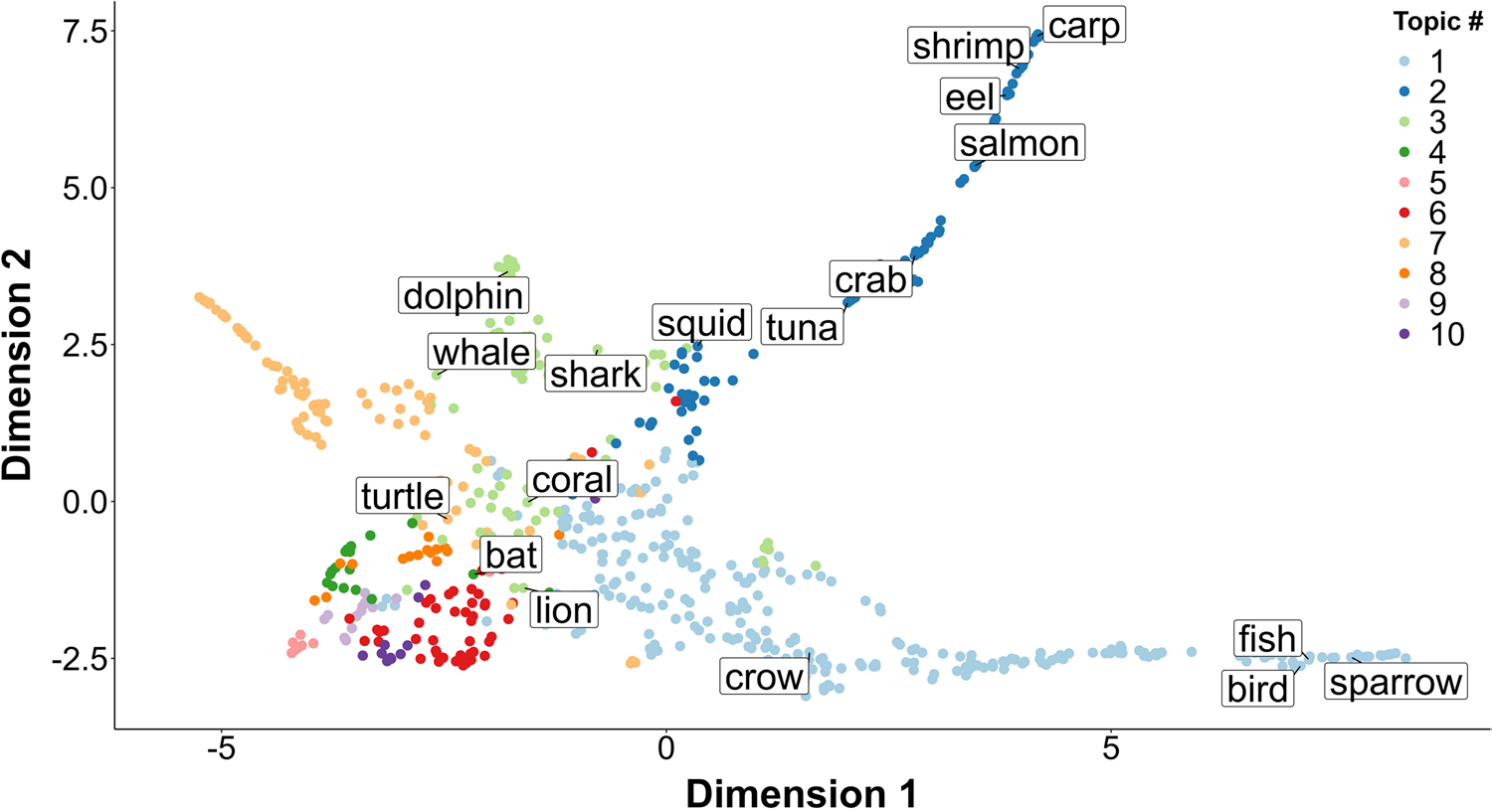
A two-dimensional illustration of the topic probabilities for animal words in our LDA model. *Note.* The colored figure is provided in the online version of this article

The results of our LDA analyses are shown in Fig. 1: a two-dimensional illustration of the topic probabilities for animal words based on our LDA model.^1^ Dimension reduction for the illustration was performed using uniform manifold approximation and projection (UMAP). UMAP is a general-purpose dimension reduction technique that preserves more of the global structure and is more computationally efficient than conventional techniques such as tSNE (McInnes et al., 2020). In the present study, we used the default hyperparameters available in the *umap* package (version 0.2.10.0; Konopka, 2023) in R. Note that in Fig. 1, for simplicity, we conveniently classified and colored each animal word corresponding to a single topic for which the word had the highest topic probability. As a result, each word is related to only one of the ten different topics in Fig. 1. Note, however, that each word had ten topic probabilities in the actual LDA model.

As shown in Fig. 1, our LDA model extracted the semantic structure of Japanese “animal” category. For instance, some words were grouped and plotted close to each other (e.g., top right in Fig. 1), suggesting that these words were predominantly related to a single topic. Critically, however, some words were not separated from others (e.g., bottom left in Fig. 1). This would reflect the desirable properties of LDA: The model allows each word to have multiple (high) topic probabilities. If a word is used in multiple topics, its topic probabilities should be high in the corresponding multiple topics. Such a word would be plotted close to other words which are related to other topics, and thus, would not be segregated from others.

As such, our LDA model allows us to quantify the semantic relationships between Japanese animal names. This also means that researchers and clinicians can use the topic probabilities of our LDA model to identify clusters in a semantic VFT. In the following, we report the results of a semantic VFT with healthy young Japanese adults.

### Verbal fluency task performance

We first calculated a conventional measure of semantic VFT, namely the total number of correct responses. In the present semantic VFT with healthy young adults, the average number of correct responses was 20.5 (*SD* = 4.9). The number of errors was also calculated. The results showed that the mean number of repetitions was 0.4 (*SD* = 0.7). On the other hand, A single wrong-category-error (i.e., intrusion) was observed (i.e., Shisa “Okinawan lion dog statue”). These results from the conventional measures are consistent with the previous VFT literature that focused on healthy young adults in other languages (e.g., Azuma, 2004; Itaguchi et al., 2022; Troyer, 2000; cf. Troyer et al., 1997).

In addition, we estimated the frequency effect for each participant and found that the mean coefficient of word frequency effect was – 0.006 log. times/s (*SD* = 0.011). This result indicates that the frequency counts of response words decreased over time. Participants initially produced high-frequency words, but later attempted to retrieve less frequent words. These patterns were also consistent with previous literature (e.g., Adams et al., 1989; Crowe, 1998; Daimon et al., 2021).

**Fig. 2:**
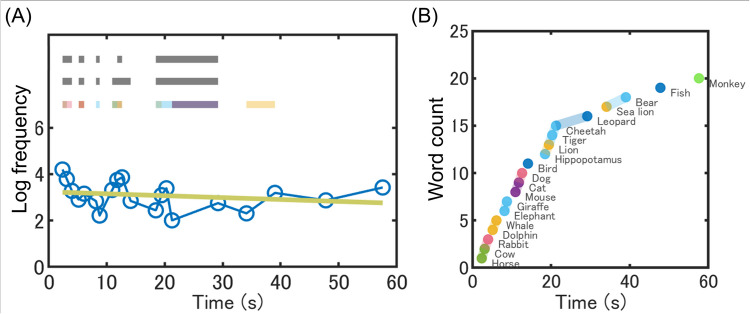
An example of semantic cluster analysis for a single participant. *Notes.* The temporal sequence of verbal fluency responses in the same participant is plotted in two ways. **A** Each response (blue circle) is plotted over time along with semantic clusters. The horizontal axis represents the timeline (from 0 to 60 s), while the vertical axis represents the logged frequency counts of each response. The yellow-green solid line represents the estimated frequency effect. The upper and lower gray bars indicate clusters detected by coder 1 and coder 2, respectively. The colored bars represent clusters detected based on our LDA model. **B** The same sequence of responses is plotted with a different vertical axis (i.e., word count). A steeper slope indicates that the participant produced animal names successively and quickly. The colors represent the highest topic probabilities in the LDA model that each response had. The colored figure is available with the online version of this article

We then performed semantic cluster analysis for each participant using two different approaches: the automatic approach using the topic probabilities in our LDA model vs. the manual approach with the human coders. An example of semantic cluster analysis is shown in Fig. 2, which illustrates the verbal fluency performance of a single participant. In the top half of Fig. 2A, each horizontal bar represents a cluster. The colored bars represent the clusters based on the LDA approach, while the gray bars represent those based on the manual approach (i.e., human coders 1 and 2). The present results show that both approaches were successful in identifying semantic clusters. However, the results from the LDA approach also revealed an additional cluster in the later part of the task (i.e., responses in 35–40 s) that was not identified by the manual approach. Figure 2B shows the results from the same participant but now highlighting the relationship between word production timing (and interval) and the LDA-based clusters (Holmlund et al., 2019).

**Fig. 3:**
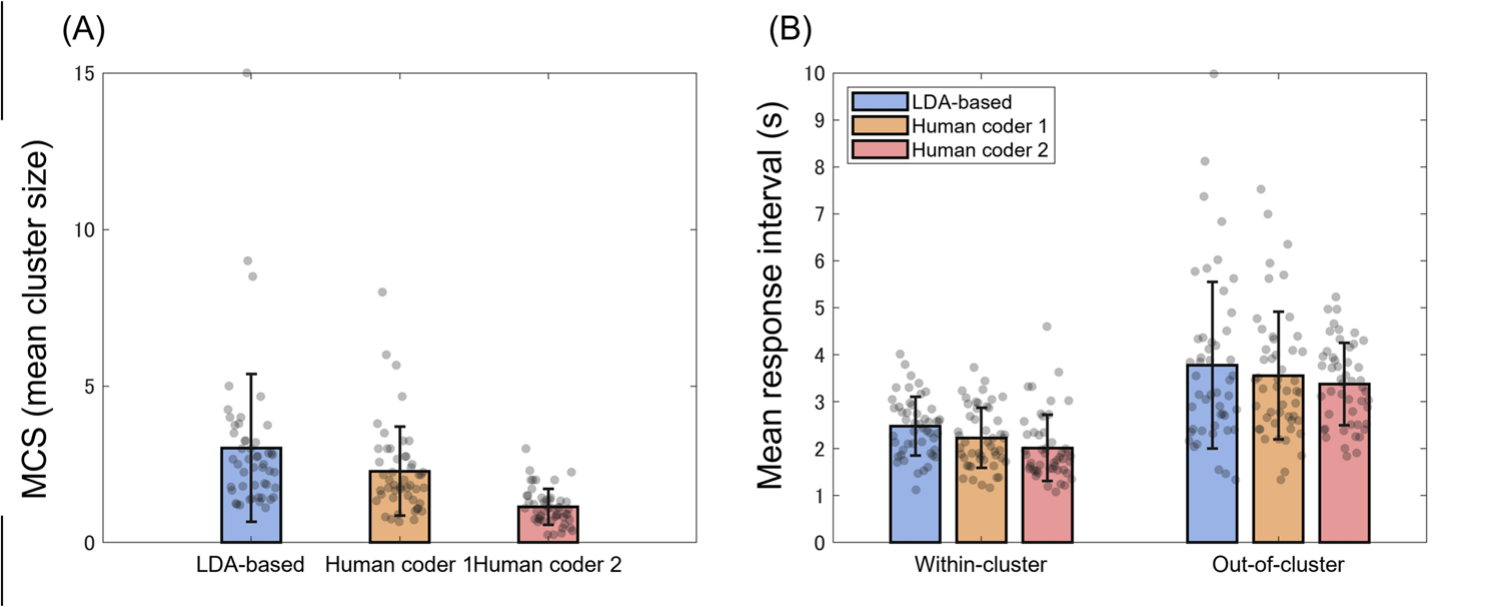
Mean cluster size **(A)** and within-cluster and out-of-cluster intervals **(B)**. *Notes.* The error bars indicate the standard deviation. The gray dots indicate individual data points

Table 2 shows the means and SDs of cluster-related indices (i.e., MCS, WCI, and OCI) for both the LDA approach and the manual approach (coders 1 and 2), and Fig. 3 also shows the results of the cluster-related indices with individual plots. Overall, the results of the LDA approach were consistent with those of the manual approach. In both approaches, semantic clusters were detected. In addition, WCI was shorter than OCI in all the coders (LDA, coder 1, and coder 2). As similar patterns of results were observed between the approaches, the present results suggest that the LDA-based semantic clustering analysis, like the human-based one, was successful in capturing the semantic structure of the Japanese “animal” category.

**Table 2:** The means of cluster-related indices in each approach

	LDA	Coder 1	Coder 2	Multiple comparisons
MCS	3.02 (2.34)	2.28 (0.57)	1.14 (0.57)	LDA > Coder 1 > Coder 2
WCI (s)	2.48 (0.62)	2.23 (0.64)	2.21 (0.70)	LDA > Coder 1 > Coder 2
OCI (s)	3.78 (1.76)	3.56 (1.34)	3.38 (0.87)	

*SD*s are in parentheses. MCI, WCI, and OCI stand for the mean cluster size, within-cluster interval (s), and out-of-cluster interval (s), respectively. Coder 1 was a professional speech therapist and coder 2 was a non-therapist. Multiple comparisons were performed using Holm’s method, and an inequality sign indicates a significant difference at the *p* <.05 level

However, we also observed that there are differences in the cluster-related indices. As shown in Table 2, all the indices (MCS, WCI, OCI) showed larger values in the LDA approach than in the manual approach. We thus tested whether the cluster-related indices were significantly different between the LDA and manual approaches. Note that the LDA approach detected only a single cluster for one participant, and hence this participant’s data was excluded from further statistical analyses.

First, we compared the MCS, a conventional clustering index, between the LDA and manual approaches by analysis of variance (ANOVA) with coding type (3 levels; LDA, coder 1, coder 2) as a single fixed factor. The results showed a significant main effect of coding type (*F*(1, 48) = 19.29, *p* <.001, η_p_^2^ =.28). Multiple comparisons with Holm’s method revealed that the MCS of human coder 1 was significantly larger than that of human coder 2 (*t*(48) = 5.88, *p* <.05). More importantly, the MCS was significantly larger for LDA clustering than for manual clustering (LDA vs. coder 1: *t*(48) = 2.07, LDA vs. coder 2: *t*(48) = 5.61, both *p*s <.05), indicating that the LDA model revealed more consecutive responses that were semantically related than the human coders did. These results are consistent with the idea that LDA can capture semantic relationships that human coders cannot (Itaguchi et al., 2022). For instance, while both human coders divided a response sequence of “dog, cat, rat, hamster, parakeet” into three clusters (i.e., “dog, cat,” “rat, hamster,” and “parakeet”), the LDA approach identified this response sequence as a single cluster where the most likely topics shifted smoothly or gradually. That is, the LDA approach first revealed that the topics with the highest topic probabilities were not common among these five animal names. Although “cat,” “rat,” and “hamster” had the highest topic probabilities for the same topic, the topic probabilities of “dog” and “parakeet” were highest for different topics. At the same time, however, the LDA model also revealed that each response was highly semantically related to multiple topics. These topics then acted as a “glue” to bind the responses into a single seamless cluster. Thus, the LDA approach makes it possible to find implicit semantic relationships between responses that might not be found by a human coder (e.g., Itaguchi et al., 2022).

Next, to confirm the validity of our LDA approach, we tested the prediction that WCI is shorter than OCI. The analysis was conducted using a two-way ANOVA with coding type (three levels; LDA, coder 1, coder 2) and interval type (two levels; WCI vs. OCI) as factors. The results showed significant main effects of coding type (*F*(2, 94) = 12.21, *p* <.001, η_p_^2^ =.21) and interval type (*F*(1, 47) = 66.92, *p* <.001, η_p_^2^ =.59), but their interaction effect was not significant (*F*(2, 94) = 0.01, *p* =.990, η_p_^2^ =.00). These results indicate that, as we predicted, WCI was significantly shorter than OCI regardless of the approaches (Fig. 3B). This analysis shows that our LDA model captured the relationship between semantic distance and processing time of words (i.e., closer semantic relationships require less processing time), further supporting the plausibility of our LDA approach to semantic clustering.

As the main effect of coding type was significant, multiple comparisons were performed using Holm’s method. The results showed that the interval measures (i.e., WCI and OCI) were significantly longer in LDA clustering than in human clustering (LDA vs. coder 1: *t*(47) = 2.42, LDA vs. coder 2:* t*(47) = 4.61, both *p*s <.05), and there was also a significant difference between the two human coders (*t*(47) = 2.85, *p* <.05). These results were somewhat unexpected, as there was no a priori reason to expect longer interval measures in the LDA approach. The longer interval measures in the LDA approach may be partly explained by the high sensitivity of the LDA models to semantic relationships between the animal words, as revealed by the MCS analysis. That is, LDA would detect weak or implicit semantic relationships between two consecutive responses whose intervals are relatively long. If consecutive responses with long intervals constitute a cluster, the WCI will increase. On the other hand, it is possible that these “relatively long” intervals are, on average, short among OCIs. If so, the OCI would also increase because relatively short intervals would not be included in the calculation. However, as these speculations are by no means definitive, future research is warranted.

Finally, we examined the clustering consistency among coders (including LDA). We calculated kappa coefficients between LDA and the two human coders (coders 1 and 2) for each participant. The mean kappa coefficient was 0.41 (*SD* = 0.23) between human coder 1 and LDA, and 0.23 (*SD* = 0.22) between human coder 2 and LDA. These low kappa coefficients indicate that the two clustering approaches each capture different aspects of the semantic structure. That is, this finding shows again that the LDA model detected semantic relationships that the human coders did not.

In addition, it is interesting to note that the mean kappa coefficient was also low 0.37 (*SD* = 0.26) between human coders 1 and 2, indicating that the consistency in clustering between human coders was not as high. This is consistent with the concern that subjective assessments of clusters by human coders may not be reproducible (Itaguchi et al., 2022; Pakhomov et al., 2015; Pakhomov & Hemmy, 2014; Zemla et al., 2020). Human coders may rely on their idiosyncratic beliefs when determining whether response words belong to the same subcategory. Consequently, it can lead to different conclusions between researchers. Specifically, our finding suggests that the backgrounds of coders affect the results of semantic clustering analysis. In the current study, human coder 1 was a professional speech therapist, whereas human coder 2 was not (a psycholinguist). The low consistency in clustering between these coders might indicate that it is difficult to directly compare the results reported by different research groups who have different theoretical knowledge or skills. Of course, this does not mean that an LDA approach is a more valid or informative way to interpret VF responses than a manual approach. Nevertheless, the present results at least provide evidence supporting the idea that computational linguistic approaches for defining semantic clusters are helpful for academic discussion and clinical practice of semantic VFTs, because they would contribute to reproducible results.

## General discussion

In previous literature, researchers have shown that computational linguistic approaches are useful when analyzing semantic clusters in semantic VFTs with alphabetic languages (Hähnel et al., 2023; Itaguchi et al., 2022; Pakhomov et al., 2015; Pakhomov & Hemmy, 2014; Rosenstein et al., 2015). However, the models proposed in these studies are not available for other languages (e.g., Japanese). In the present study, we constructed a Japanese LDA model for clustering analyses in the semantic VFT of the “animal” category. The model assigned topic probabilities to animal names, which quantified the semantic relatedness between animal names. Inspection of a two-dimensional illustration (Fig. 1) showed that our LDA model extracted the semantic structure of the Japanese “animal” category, enabling us to identify semantic clusters automatically and objectively.

In the semantic VFT with healthy young Japanese adults, the overall patterns of the results were consistent between the automatic approach with our LDA model and the manual approach with the human coders. First, like the manual approach, the automatic approach identified clusters in the semantic VFT of the “animal” category, as was reflected in the MCS. Second, our prediction regarding the response interval was confirmed in both approaches: WCI was shorter than OCI, indicating that semantically related words were processed faster than semantically unrelated words. These results indicate that the LDA-based approach is also useful in the semantic VFT with Japanese, as previous literature has shown in the VFT with alphabetic languages (e.g., Hähnel et al., 2023; Itaguchi et al., 2022; Pakhomov et al., 2015; Pakhomov & Hemmy, 2014; Rosenstein et al., 2015).

That we observed shorter WCI than OCI is particularly important because it is consistent with classical network models of semantic representations, such as the spreading activation theory (Collins & Loftus, 1975). It is generally assumed that semantically related words are closely represented, and thus, activation spreads rapidly. Under this assumption, we predicted that response time intervals within a cluster would be shorter than those outside a cluster because semantic relatedness between response words should be higher in a cluster than outside a cluster. The results confirmed this prediction in both the LDA and manual approaches. To the best of our knowledge, our finding is the first empirical demonstration of the relationship between response intervals and clusters in semantic VFTs. Furthermore, the shorter WCI compared to OCI is consistent with psycholinguistic findings such as semantic priming effects (Neely, 1991), as both reflect that semantically related words are processed faster than semantically unrelated words. Taken together, the current results indicate that our LDA model (as well as the manual clustering) extracted the structure of the Japanese “animal” category, providing further support for the usefulness of the computational semantic clustering approach.

The current results also revealed differences between the automatic and manual approaches. First, a larger MCS was observed for the automatic approach than for the manual approach. Second, both response interval measures (i.e., WCI and OCI) were longer for the automatic approach than for the manual approach. Finally, we observed a low kappa coefficient between the automatic and manual clustering results. These results suggest that our LDA model and the human coders identified clusters in different ways. Specifically, LDA models may be more sensitive to implicit semantic relationships than human coders. This possibility has already been raised in previous literature. In Itaguchi et al. (2022), only LDA analysis revealed that intrusion errors produced by patients with mild AD were conceptually related to the “animal” category, suggesting that some semantic relationships are still preserved in that population. Further research is warranted to investigate whether the LDA model is indeed more sensitive than human coders. For instance, it is important to investigate whether intrusion errors in AD are conceptually related to the *Japanese* “animal” category.

It is worth noting that we observed a significant frequency effect in the semantic VFT (e.g., Adams et al., 1989; Crowe, 1998; Daimon et al., 2021). The participants tended to produce higher-frequency words early in the task, whereas they produced lower-frequency words later. While word frequency effects have received relatively little attention in the literature on VFTs (Daimon et al., 2021), considerable effort has been devoted to examining word frequency effects in psycholinguistic literature. Indeed, the effect of word frequency is found to be particularly reliable and large in the word recognition and production literature (Brysbaert et al., 2011; Loewenstein et al., 2012; Monsell et al., 1989; Rubenstein et al., 1970). Word frequency effects are generally assumed to arise in the process of selecting a lexical representation (i.e., lexical access), where high-frequency words are activated faster and more strongly than low-frequency words (Levelt et al., 1999; McClelland & Rumelhart, 1981). As the VFTs are assumed to involve the process of lexical access (e.g., Carpenter et al., 2020; Shao et al., 2014; Tröster et al., 1998), further research of word frequency effects is needed to investigate the nature of lexical access in VFTs.

Before concluding, we will discuss the limitations of the current study, namely, our limited participants. In the current study, we focused on healthy young adults only. Specifically, we recruited university students at Waseda University. As this university is regarded as one of the most prestigious private ones in Japan, it is not reasonable to assume that our participants were representative of Japanese populations. Previous research has shown that the task performance of the VFT is influenced by various demographic variables such as age, gender, educational level, and working memory (Acevedo et al., 2000; Azuma, 2004; Chan & Poon, 1999; Kempler et al., 1998; Kozora & Cullum, 1995; McDowd et al., 2011; Troyer, 2000). Thus, it is essential in future research to recruit participants from broader populations and investigate the effects of these variables on the semantic VFTs with Japanese. Furthermore, it is also critical to extend the present findings to clinical populations. As noted in the Introduction, performance of the semantic VFT is related to neurological disorders such as AD (Fagundo et al., 2008; Ober et al., 1986; Rosen, 1980; Tröster et al., 1989; Troyer et al. 1998a, b; but see Raoux et al., 2008), Parkinson’s disease (Tröster et al., 1998; Troyer et al. 1998b), Huntington’s disease (Rich et al., 1999), multiple sclerosis (Tröster et al., 1998), schizophrenia (Robert et al., 1998), stroke (Engstad et al., 2003; Schmidt et al., 2019), and traumatic brain injury (Henry & Crawford, 2004; Woods et al., 2016). However, there is still no internationally available normative dataset of Japanese participants and patients. Given the importance of normative datasets for their clinical use (Villalobos et al., 2023), it is important to collect data from a larger group of Japanese participants to further increase the utility of the current LDA model and normative dataset.

## Conclusion

In conclusion, the present study provides a Japanese LDA model. We showed that our LDA model can be used for semantic clustering analysis of healthy Japanese young adults. Using this model, researchers and clinicians can obtain consistent and objective results across participants or patients, reduce time and personnel costs (especially in clinical settings), and thus increase the efficiency of assessments based on semantic VFTs. We hope that the present study will promote the use of a computational linguistic approach for clustering analysis of semantic VFTs in the Japanese language.

## Supplementary Information

Below is the link to the electronic supplementary material.

Supplementary file1 (CSV 73 KB)

## Data Availability

The LDA model and VFT experimental data are available at https://osf.io/pukax/.

## Footnotes

1 For interested readers, we uploaded a three-dimensional illustration of our LDA model at https://osf.io/pukax/

## Code availability

The codes for analyzing the VFT data are available at https://osf.io/pukax/.

## References

[CR1] Acevedo, A., Loewenstein, D. A., Barker, W. W., Harwood, D. G., Luis, C., Bravo, M., Hurwitz, D. A., Aguero, H., Greenfield, L., & Duara, R. (2000). Category fluency test: Normative data for English- and Spanish-speaking elderly. *Journal of the International Neuropsychological Society,**6*(7), 760–769. 10.1017/S1355617700677032 11105466 10.1017/s1355617700677032

[CR2] Adams, M. L., Reich, A. R., & Flowers, C. R. (1989). Verbal fluency characteristics of normal and aphasic speakers. *Journal of Speech, Language, and Hearing Research,**32*(4), 871–879. 10.1044/jshr.3204.871 10.1044/jshr.3204.871 2601317

[CR3] Ardila, A. (2020). A cross-linguistic comparison of category verbal fluency test (ANIMALS): A systematic review. *Archives of Clinical Neuropsychology,**35*(2), 213–225. 10.1093/arclin/acz060 31813955 10.1093/arclin/acz060

[CR4] Audacity Team (2021). Audacity(R): Free audio editor and recorder (Version 3.0) [Computer software]. https://audacityteam.org/

[CR5] Auriacombe, S., Lechevallier, N., Amieva, H., Harston, S., Raoux, N., & Dartigues, J.-F. (2006). A longitudinal study of quantitative and qualitative features of category verbal fluency in incident Alzheimer’s disease subjects: Results from the PAQUID study. *Dementia and Geriatric Cognitive Disorders,**21*(4), 260–266. 10.1159/000091407 16465054 10.1159/000091407

[CR6] Azuma, T. (2004). Working memory and perseveration in verbal fluency. *Neuropsychology,**18*(1), 69–77. 10.1037/0894-4105.18.1.69 14744189 10.1037/0894-4105.18.1.69

[CR7] Blei, D. M., Ng, A. Y., & Jordan, M. I. (2003). Latent Dirichlet allocation. *Journal of Machine Learning Research,**3*, 993–1022. 10.5555/944919.944937

[CR8] Bose, A., Wood, R., & Kiran, S. (2017). Semantic fluency in aphasia: Clustering and switching in the course of 1 minute. *International Journal of Language & Communication Disorders,**52*(3), 334–345. 10.1111/1460-6984.12276 27767243 10.1111/1460-6984.12276

[CR9] Brysbaert, M., Buchmeier, M., Conrad, M., Jacobs, A. M., Bölte, J., & Böhl, A. (2011). The word frequency effect: A review of recent developments and implications for the choice of frequency estimates in German. *Experimental Psychology,**58*(5), 412–424. 10.1027/1618-3169/a000123 21768069 10.1027/1618-3169/a000123

[CR10] Bushnell, J., Svaldi, D., Ayers, M. R., Gao, S., Unverzagt, F., Gaizo, J. D., ..., & Clark, D. G. (2022). A comparison of techniques for deriving clustering and switching scores from verbal fluency word lists. *Frontiers in Psychology, 13*, 743557. 10.3389/fpsyg.2022.743557 10.3389/fpsyg.2022.743557 PMC9518694 36186334

[CR11] Carpenter, E., Rao, L., Peñaloza, C., & Kiran, S. (2020). Verbal fluency as a measure of lexical access and cognitive control in bilingual persons with aphasia. *Aphasiology*. 10.1080/02687038.2020.1759774 10.1080/02687038.2020.1759774 PMC8341392 34366537

[CR12] Chan, A. S., & Poon, M. W. (1999). Performance of 7- to 95-year-old individuals in a Chinese version of the category fluency test. *Journal of the International Neuropsychological Society,**5*(6), 525–533. 10.1017/S135561779956606X 10561933 10.1017/s135561779956606x

[CR13] Collins, A. M., & Loftus, E. F. (1975). A spreading-activation theory of semantic processing. *Psychological Review,**82*(6), 407–428.

[CR14] Crowe, S. F. (1998). Decrease in performance on the verbal fluency test as a function of time: Evaluation in a young healthy sample. *Journal of Clinical and Experimental Neuropsychology,**20*(3), 391–401. 10.1076/jcen.20.3.391.810 9845165 10.1076/jcen.20.3.391.810

[CR15] Daimon, S., Noto, S., & Itaguchi, Y. (2021). Kurikaeshi Jisshi Shita Gengo Ryuuchousei Kadai Ni Taisuru Jikan Jouhou To Hindo Jouhou Wo Mochiita Teiryouteki Kaiseki [Quantitative analyses on the performance of repeated verbal fluency tests using time and word frequency information]. *Higher Brain Function Research,**41*(4), 21–30. 10.2496/hbfr.41.387

[CR16] Deerwester, S., Dumais, S. T., Furnas, G. W., Landauer, T. K., & Harshman, R. (1990). Indexing by latent semantic analysis. *Journal of the American Society for Information Science,**41*(6), 391–407. 10.1002/(SICI)1097-4571(199009)41:6<391::AID-ASI1>3.0.CO;2-9

[CR17] Devlin, J., Chang, M.-W., Lee, K., & Toutanova, K. (2019). BERT: Pre-training of deep bidirectional transformers for language understanding. In J. Burstein, C. Doran, & T. Solorio (Eds.), *NAACL-HLT* (Vol. 1, pp. 4171–4186). 10.48550/arXiv.1810.04805

[CR18] Engstad, T., Almkvist, O., Viitanen, M., & Arnesen, E. (2003). Impaired motor speed, visuospatial episodic memory and verbal fluency characterize cognition in long-term stroke survivors: The Tromsø study. *Neuroepidemiology,**22*(6), 326–331. 10.1159/000072921 14557682 10.1159/000072921

[CR19] Fagundo, A. B., López, S., Romero, M., Guarch, J., Marcos, T., & Salamero, M. (2008). Clustering and switching in semantic fluency: Predictors of the development of Alzheimer’s disease. *International Journal of Geriatric Psychiatry,**23*(10), 1007–1013. 10.1002/gps.2025 18416452 10.1002/gps.2025

[CR20] Gomez, R., & White, D. (2006). Using verbal fluency to detect very mild dementia of the Alzheimer type. *Archives of Clinical Neuropsychology,**21*(8), 771–775. 10.1016/j.acn.2006.06.012 17011743 10.1016/j.acn.2006.06.012

[CR21] Griffiths, T. L., & Steyvers, M. (2004). Finding scientific topics. *Proceedings of the National Academy of Sciences, 101*(suppl_1), 5228–5235. 10.1073/pnas.0307752101 10.1073/pnas.0307752101 PMC387300 14872004

[CR22] Gruenewald, P. J., & Lockhead, G. R. (1980). The free recall of category examples. *Journal of Experimental Psychology: Human Learning and Memory,**6*(3), 225–240. 10.1037/0278-7393.6.3.225

[CR23] Hähnel, T., Feige, T., Kunze, J., Epler, A., Frank, A., Bendig, J., Schnalke, N., Wolz, M., Themann, P., & Falkenburger, B. (2023). A semantic relatedness model for the automatic cluster analysis of phonematic and semantic verbal fluency tasks performed by people with Parkinson disease: Prospective multicenter study. *JMIR Neurotechnology,**2*(1), e46021. 10.2196/46021 10.2196/46021 PMC12671300 41341984

[CR24] Henry, J. D., & Crawford, J. R. (2004). A meta-analytic review of verbal fluency performance in patients with traumatic brain injury. *Neuropsychology,**18*(4), 621–628. 10.1037/0894-4105.18.4.621 15506829 10.1037/0894-4105.18.4.621

[CR25] Henry, J. D., Crawford, J. R., & Phillips, L. H. (2004). Verbal fluency performance in dementia of the Alzheimer’s type: A meta-analysis. *Neuropsychologia,**42*(9), 1212–1222. 10.1016/j.neuropsychologia.2004.02.001 15178173 10.1016/j.neuropsychologia.2004.02.001

[CR26] Hofmann, M. J., Remus, S., Biemann, C., Radach, R., & Kuchinke, L. (2022). Language models explain word reading times better than empirical predictability. *Frontiers in Artificial Intelligence*, *4*. 10.3389/frai.2021.730570 10.3389/frai.2021.730570 PMC8847793 35187472

[CR27] Holmlund, T. B., Cheng, J., Foltz, P. W., Cohen, A. S., & Elvevåg, B. (2019). Updating verbal fluency analysis for the 21st century: Applications for psychiatry. *Psychiatry Research,**273*, 767–769. 10.1016/j.psychres.2019.02.014 31207864 10.1016/j.psychres.2019.02.014

[CR28] Itaguchi, Y., Castro-Chavira, S. A., Waterloo, K., Johnsen, S. H., & Rodríguez-Aranda, C. (2022). Evaluation of error production in animal fluency and its relationship to frontal tracts in normal aging and mild Alzheimer’s disease: A combined LDA and time-course analysis investigation. *Frontiers in Aging Neuroscience,**13*, 710938. 10.3389/fnagi.2021.710938 35095462 10.3389/fnagi.2021.710938 PMC8790484

[CR29] Jelodar, H., Wang, Y., Yuan, C., Feng, X., Jiang, X., Li, Y., & Zhao, L. (2019). Latent Dirichlet allocation (LDA) and topic modeling: models, applications, a survey. *Multimedia Tools and Applications,**78*, 15169–15211. 10.1007/s11042-018-6894-4

[CR30] Kempler, D., Teng, E. L., Dick, M., Taussig, I. M., & Davis, D. S. (1998). The effects of age, education, and ethnicity on verbal fluency. *Journal of the International Neuropsychological Society,**4*(6), 531–538. 10.1017/S1355617798466013 10050357 10.1017/s1355617798466013

[CR31] König, A., Linz, N., Tröger, J., Wolters, M., Alexandersson, J., & Robert, P. (2018). Fully automatic speech-based analysis of the semantic verbal fluency task. *Dementia and Geriatric Cognitive Disorders,**45*(3–4), 198–209. 10.1159/000487852 29886493 10.1159/000487852

[CR32] Konopka, T. (2023). umap: Uniform Manifold Approximation and Projection (Version 0.2.10.0) [Computer software]. https://cran.r-project.org/package=umap

[CR33] Kozora, E., & Cullum, C. M. (1995). Generative naming in normal aging: Total output and qualitative changes using phonemic and semantic constraints. *The Clinical Neuropsychologist,**9*(4), 313–320. 10.1080/13854049508400495

[CR34] Landauer, T. K., & Dumais, S. T. (1997). A solution to Plato’s problem: The latent semantic analysis theory of acquisition, induction, and representation of knowledge. *Psychological Review,**104*(2), 211–240. 10.1037/0033-295X.104.2.211

[CR35] Levelt, W. J. M., Roelofs, A., & Meyer, A. S. (1999). A theory of lexical access in speech production. *Behavioral And Brain Sciences.,**22*(1), 1–38. 11301520 10.1017/s0140525x99001776

[CR36] Lezak, M. D. (2005). *Neuropsychological assessment* (Shinkei-shinrigaku shusei. M. Mimura, T. Muramatsu, H. Kashima, Trans.; 3rd edn.). Sozo-Shupp. Oxford University Press.

[CR37] Loewenstein, D. A., Greig, M. T., Schinka, J. A., Barker, W., Shen, Q., Potter, E., Raj, A., Brooks, L., Varon, D., Schoenberg, M., Banko, J., Potter, H., & Duara, R. (2012). An investigation of PreMCI: Subtypes and longitudinal outcomes. *Alzheimer’s & Dementia,**8*(3), 172–179. 10.1016/j.jalz.2011.03.002 10.1016/j.jalz.2011.03.002 PMC3341845 22546351

[CR38] McClelland, J. L., & Rumelhart, D. E. (1981). An interactive activation model of context effects in letter perception: I. An account of basic findings. *Psychological Review,**88*(5), 375–407. 10.1037/0033-295X.88.5.375 7058229

[CR39] McDowd, J., Hoffman, L., Rozek, E., Lyons, K. E., Pahwa, R., Burns, J., & Kemper, S. (2011). Understanding verbal fluency in healthy aging, Alzheimer’s disease, and Parkinson’s disease. *Neuropsychology,**25*(2), 210–225. 10.1037/a0021531 21381827 10.1037/a0021531

[CR40] McInnes, L., Healy, J., & Melville, J. (2020). *UMAP: Uniform Manifold Approximation and Projection for Dimension Reduction* (arXiv:1802.03426). arXiv. http://arxiv.org/abs/1802.03426

[CR41] Mikolov, T., Corrado, G., Chen, K., & Dean, J. (2013a). Efficient estimation of word representations in vector space. In *1st International Conference on Learning Representations, ICLR 2013* (pp. 1–12). https://api.semanticscholar.org/CorpusID:5959482

[CR42] Mikolov, T., Sutskever, I., Chen, K., Corrado, G., & Dean, J. (2013b). Distributed representations of words and phrases and their compositionality. In *NIPS'13: Proceedings of the 26th International Conference on Neural Information Processing Systems* (Vol. 2, pp. 3111–3119). 10.48550/arXiv.1310.4546

[CR43] Mitsuto, R., Nishikoori, T., Tatsukawa, K., Hashimoto, Y., & Miyatani, M. (2019). Arutsuhaimaa-byoo To Keido Ninchi Syougai Ni Okeru Gengo Ryuuchousei Kadai No Shitsuteki Kentou [Qualitative analysis of verbal fluency tasks in Alzheimer’s disease and mild cognitive impairment]. *Higher Brain Function Research,**39*(1), 18–27. 10.2496/hbfr.39.18

[CR44] Mollo, G., Karapanagiotidis, T., Bernhardt, B. C., Murphy, C. E., Smallwood, J., & Jefferies, E. (2016). An individual-differences analysis of the neurocognitive architecture of the semantic system at rest. *Brain and Cognition,**109*, 112–123. 10.1016/j.bandc.2016.07.003 27662589 10.1016/j.bandc.2016.07.003 PMC5090046

[CR45] Monsell, S., Doyle, M. C., & Haggard, P. N. (1989). Effects of frequency on visual word recognition tasks: Where are they? *Journal of Experimental Psychology: General,**118*(1), 43–71. 10.1037/0096-3445.118.1.43 2522506 10.1037//0096-3445.118.1.43

[CR46] Neely, J. H. (1991). Semantic priming effects in visual word recognition: A selective review of current findings and theories. *Basic Processes in Reading. *Routledge.

[CR47] Ober, B. A., Dronkers, N. F., Koss, E., Delis, D. C., & Friedland, R. P. (1986). Retrieval from semantic memory in Alzheimer-type dementia. *Journal of Clinical and Experimental Neuropsychology,**8*(1), 75–92. 10.1080/01688638608401298 3944246 10.1080/01688638608401298

[CR48] Pakhomov, S. V. S., & Hemmy, L. S. (2014). A computational linguistic measure of clustering behavior on semantic verbal fluency task predicts risk of future dementia in the Nun Study. *Cortex,**55*, 97–106. 10.1016/j.cortex.2013.05.009 23845236 10.1016/j.cortex.2013.05.009 PMC4402214

[CR49] Pakhomov, S. V. S., Jones, D. T., & Knopman, D. S. (2015). Language networks associated with computerized semantic indices. *NeuroImage,**104*, 125–137. 10.1016/j.neuroimage.2014.10.008 25315785 10.1016/j.neuroimage.2014.10.008 PMC4402216

[CR50] Panichella, A. (2021). A systematic comparison of search-based approaches for LDA hyperparameter tuning. *Information and Software Technology,**130*, Article 106411. 10.1016/j.infsof.2020.106411

[CR51] Pennington, J., Socher, R., & Manning, C. (2014). GloVe: Global vectors for word representation. In *Association for Computational Linguistics Proceedings of the 2014 Conference on Empirical Methods in Natural Language Processing (EMNLP)*. 10.3115/v1/D14-1162

[CR52] Pereira, F., Botvinick, M., & Detre, G. (2013). Using Wikipedia to learn semantic feature representations of concrete concepts in neuroimaging experiments. *Artificial Intelligence,**194*, 240–252. 10.1016/j.artint.2012.06.005 23243317 10.1016/j.artint.2012.06.005 PMC3519435

[CR53] Raoux, N., Amieva, H., Le Goff, M., Auriacombe, S., Carcaillon, L., Letenneur, L., & Dartigues, J.-F. (2008). Clustering and switching processes in semantic verbal fluency in the course of Alzheimer’s disease subjects: Results from the PAQUID longitudinal study. *Cortex,**44*(9), 1188–1196. 10.1016/j.cortex.2007.08.019 18761132 10.1016/j.cortex.2007.08.019

[CR54] Rich, J. B., Troyer, A. K., Bylsma, F. W., & Brandt, J. (1999). Longitudinal analysis of phonemic clustering and switching during word-list generation in Huntington’s disease. *Neuropsychology,**13*(4), 525–531. 10.1037/0894-4105.13.4.525 10527060 10.1037//0894-4105.13.4.525

[CR55] Robert, P. H., Lafont, V., Medecin, I., Berthet, L., Thauby, S., Baudu, C., & Darcourt, G. (1998). Clustering and switching strategies in verbal fluency tasks: Comparison between schizophrenics and healthy adults. *Journal of the International Neuropsychological Society,**4*(6), 539–546. 10.1017/S1355617798466025 10050358 10.1017/s1355617798466025

[CR56] Rofes, A., de Aguiar, V., Jonkers, R., Oh, S. J., DeDe, G., & Sung, J. E. (2020). What drives task performance during animal fluency in people with Alzheimer’s disease? *Frontiers in Psychology*, *11*. 10.3389/fpsyg.2020.01485 10.3389/fpsyg.2020.01485 PMC7388773 32774312

[CR57] Rosen, W. G. (1980). Verbal fluency in aging and dementia. *Journal of Clinical Neuropsychology,**2*(2), 135–146. 10.1080/01688638008403788

[CR58] Rubenstein, H., Garfield, L., & Millikan, J. A. (1970). Homographic entries in the internal lexicon. *Journal of Verbal Learning and Verbal Behavior,**9*(5), 487–494. 10.1016/S0022-5371(70)80091-3

[CR59] Rosenstein, M., Foltz, P., Vaskinn, A., & Elvevåg, B. (2015). Practical issues in developing semantic frameworks for the analysis of verbal fluency data: A Norwegian data case study. *Proceedings of the 2nd Workshop on Computational Linguistics and Clinical Psychology: From Linguistic Signal to Clinical Reality* (pp. 124–133). Association for Computational Linguistics. 10.3115/v1/W15-1215

[CR60] Schmidt, C. S. M., Nitschke, K., Bormann, T., Römer, P., Kümmerer, D., Martin, M., Umarova, R. M., Leonhart, R., Egger, K., Dressing, A., Musso, M., Willmes, K., Weiller, C., & Kaller, C. P. (2019). Dissociating frontal and temporal correlates of phonological and semantic fluency in a large sample of left hemisphere stroke patients. *NeuroImage: Clinical,**23*, 101840. 10.1016/j.nicl.2019.101840 31108458 10.1016/j.nicl.2019.101840 PMC6526291

[CR61] Shao, Z., Janse, E., Visser, K., & Meyer, A. S. (2014). What do verbal fluency tasks measure? Predictors of verbal fluency performance in older adults. *Frontiers in Psychology*, *5*. 10.3389/fpsyg.2014.00772 10.3389/fpsyg.2014.00772 PMC4106453 25101034

[CR62] Snowdon, D. A. (1997). Aging and Alzheimer’s disease: Lessons from the Nun Study1. *The Gerontologist,**37*(2), 150–156. 10.1093/geront/37.2.150 9127971 10.1093/geront/37.2.150

[CR63] Stan Development Team (2018). RStan: the R interface to Stan (Version 2.18.2) [Computer software]. https://cran.r-project.org/package=rstan

[CR64] Steyvers, M., Griffiths, T. L., & Dennis, S. (2006). Probabilistic inference in human semantic memory. *Trends in Cognitive Sciences,**10*(7), 327–334. 10.1016/j.tics.2006.05.005 16793324 10.1016/j.tics.2006.05.005

[CR65] Tröster, A. I., Salmon, D. P., McCullough, D., & Butters, N. (1989). A comparison of the category fluency deficits associated with Alzheimer’s and Huntington’s disease. *Brain and Language,**37*(3), 500–513. 10.1016/0093-934X(89)90032-1 2529947 10.1016/0093-934x(89)90032-1

[CR66] Tröster, A. I., Fields, J. A., Testa, J. A., Paul, R. H., Blanco, C. R., Hames, K. A., Salmon, D. P., & Beatty, W. W. (1998). Cortical and subcortical influences on clustering and switching in the performance of verbal fluency tasks. *Neuropsychologia,**36*(4), 295–304. 10.1016/S0028-3932(97)00153-X 9665640 10.1016/s0028-3932(97)00153-x

[CR67] Troyer, A. K. (2000). Normative data for clustering and switching on verbal fluency tasks. *Journal of Clinical and Experimental Neuropsychology,**22*(3), 370–378. 10.1076/1380-3395(200006)22:3;1-V;FT370 10855044 10.1076/1380-3395(200006)22:3;1-V;FT370

[CR68] Troyer, A. K., Moscovitch, M., & Winocur, G. (1997). Clustering and switching as two components of verbal fluency: Evidence from younger and older healthy adults. *Neuropsychology,**11*(1), 138–146. 10.1037/0894-4105.11.1.138 9055277 10.1037//0894-4105.11.1.138

[CR69] Troyer, A. K., Moscovitch, M., Winocur, G., Alexander, M. P., & Stuss, D. (1998a). Clustering and switching on verbal fluency: The effects of focal frontal- and temporal-lobe lesions. *Neuropsychologia,**36*(6), 499–504. 10.1016/S0028-3932(97)00152-8 9705059 10.1016/s0028-3932(97)00152-8

[CR70] Troyer, A. K., Moscovitch, M., Winocur, G., Leach, L., & Freedman, M. (1998b). Clustering and switching on verbal fluency tests in Alzheimer’s and Parkinson’s disease. *Journal of the International Neuropsychological Society,**4*(2), 137–143. 10.1017/S1355617798001374 9529823 10.1017/s1355617798001374

[CR71] Unsworth, N., Spillers, G. J., & Brewer, G. A. (2011). Variation in verbal fluency: A latent variable analysis of clustering, switching, and overall performance. *Quarterly Journal of Experimental Psychology,**64*(3), 447–466. 10.1080/17470218.2010.505292 10.1080/17470218.2010.505292 20839136

[CR72] Villalobos, D., Povedano-Montero, J., Fernández, S., López-Muñoz, F., Pacios, J., & del Río, D. (2022). Scientific research on verbal fluency tests: A bibliometric analysis. *Journal of Neurolinguistics,**63*, 101082. 10.1016/j.jneuroling.2022.101082

[CR73] Villalobos, D., Torres-Simón, L., Pacios, J., Paúl, N., & del Río, D. (2023). A systematic review of normative data for verbal fluency test in different languages. *Neuropsychology Review,**33*(4), 733–764. 10.1007/s11065-022-09549-0 36098929 10.1007/s11065-022-09549-0

[CR74] Woods, D. L., Wyma, J. M., Herron, T. J., & Yund, E. W. (2016). Computerized analysis of verbal fluency: Normative data and the effects of repeated testing, simulated malingering, and traumatic brain injury. *PLOS ONE,**11*(12), e0166439. 10.1371/journal.pone.0166439 27936001 10.1371/journal.pone.0166439 PMC5147824

[CR75] Zemla, J. C., Cao, K., Mueller, K. D., & Austerweil, J. L. (2020). SNAFU: The semantic network and fluency utility. *Behavior Research Methods,**52*(4), 1681–1699. 10.3758/s13428-019-01343-w 32128696 10.3758/s13428-019-01343-w PMC7406526

